# Relationship of Clinical and Pathologic Nodal Staging in Locally Advanced Breast Cancer: Current Controversies in Daily Practice?

**DOI:** 10.14740/jocmr1908w

**Published:** 2014-09-09

**Authors:** Francesca De Felice, Daniela Musio, Nadia Bulzonetti, Nicola Raffetto, Vincenzo Tombolini

**Affiliations:** a Cattedra di Radioterapia, Dipartimento di Scienze Radiologiche Oncologiche e Anatomo-Patologiche, “Sapienza” University of Rome, Rome, Italy; b Fondazione Spencer-Lorillard, Rome, Italy

**Keywords:** Breast cancer, Lymph nodes, Staging, Clinical stage, Pathologic

## Abstract

Systemic neo-adjuvant therapy plays a primary role in the management of locally advanced breast cancer. Without having any negative effect in overall survival, induction chemotherapy potentially assures a surgery approach in unresectable disease or a conservative treatment in technically resectable disease and acts on a well-vascularized tumor bed, without the modifications induced by surgery. A specific issue has a central function in the neo-adjuvant setting: lymph nodes status. It still represents one of the strongest predictors of long-term prognosis in breast cancer. The discussion of regional radiation therapy should be a matter of debate, especially in a pathological complete response. Currently, the indication for radiotherapy is based on the clinical stage before the surgery, even for the irradiation of the loco-regional lymph nodes. Regardless of pathological down-staging, radiation therapy is accepted as standard adjuvant treatment in locally advanced breast cancer.

## Introduction

Breast cancer is the most common cancer in women worldwide, with 232,340 estimated new cases in 2013 [1]. The rate of patients with breast cancer has improved in recent decades, due to screening-programs, and accurate staging has become important in the clinical management of these patients. The usual staging consists of mammography/ultrasonography followed by, after positive biopsy, conventional imaging with chest radiograph, and, in advance stages, bone scan and computed tomography of abdomen-pelvis to detect distant metastases. Magnetic resonance imaging of breast is indicated for differential diagnosis in those selected cases of questionable findings on physical examination, mammography and ultrasound [2].

Locally advanced breast cancer is defined by the 2013 National Comprehensive Cancer Network as stage IIIA and IIIB disease (any TN2 and any stage ≥ T3N1) [3]. In the era of personalized medicine, the role of systemic therapy to guarantee an additional benefit of conservative surgery is well defined, in this subgroup of patients. But, a clinical response, both partial or complete, does not influence radiation therapy (RT) indication [4]. Nevertheless, the role of clinical nodal stage versus pathological nodal stage remains an unresolved issue. This consideration is important because, although modern multidisciplinary approach has significantly improved the efficacy of treatment, adjuvant RT still carries the sterilization of residual tumor in primary disease and regional lymph nodes. It is our objective that this analysis will raise clinicians in defining a precise clinical extent of disease.

## Overview

The question of correctly staging clinical nodal disease at diagnosis is an unresolved problem with which radiation oncologist is usually confronted.

The current standard treatment approach for patients presenting with locally advanced breast cancer is neo-adjuvant chemotherapy. Approximately 80-90% of patients will show partial or complete clinical response to preoperative chemotherapy [5, 6], and they become candidates for a more conservative surgery or if with inoperable disease at onset, for a surgical procedure. Regardless of the response to preoperative chemotherapy, both partial or complete, adjuvant RT should be considered for these patients [4].

An accurate clinical stage disease is paramount and it needs to be optimized. A specific issue plays a central role in the neo-adjuvant setting: lymph nodes status. Response to neo-adjuvant chemotherapy could translate in a higher probability of conservative surgery, but it is not related to guide decisions regarding RT indication. Therefore, it is mandatory to evaluate carefully the initial extent of disease in all patients and to assign a clinical stage before any treatment is begun. Specific aspects of both primary tumor and nodal clinical staging pre-surgery in breast cancer may have implications for treatments given later in the course of multimodality therapy.

## Nodal Stage System

Current literature suggests that the TNM stage system is the standard classification of breast cancer based on the anatomical extent of the disease. The lymph node (N) factor essentially evaluated the absence/presence and the extent of regional lymph node metastases. Both clinical and pathological staging systems have been established for nodal disease. For practicality sake only N1 and N2 stages are reported. Details are shown in Table 1. Clinical N1 disease signifies metastases to movable ipsilateral level I, II axillary lymph node(s). Clinical N2 disease signifies either involved axillary lymph nodes that are fixed to one another or to other surrounding structures (N2a) or involved internal mammary lymph nodes in the absence of clinically evident axillary lymph node disease (N2b), as determined by physical examination or imaging studies. Pathological N1 disease represents micro-metastases (pN1mic), or metastases in 1 - 3 axillary lymph nodes (pN1a), or/and in internal mammary nodes with metastases (pN1b/pN1c) detected by sentinel lymph node biopsy but not clinically detected. Pathological N2 disease represents involvement of 4 - 9 axillary lymph nodes with at least one tumor deposit measuring over 2.0 mm (N2a) or clinical involvement of internal mammary lymph nodes with pathologically negative axillary lymph nodes (N2b) [7].

**Table 1:** Regional Lymph Nodes (N)

Clinical	
Nx	Regional lymph nodes cannot be assessed
N0	No regional lymph node metastasis
N1	Metastases to movable ipsilateral level I, II axillary lymph node(s)
N2	Metastases in ipsilateral level I, II axillary lymph nodes that are clinically fixed or matted; or in clinically detected ipsilateral internal mammary nodes in the absence of clinically evident axillary lymph node metastases
N2a	Metastases in ipsilateral level I, II axillary lymph nodes fixed to one another (matted) or to other structures
N2b	Metastases only in clinically detected ipsilateral internal mammary nodes and in the absence of clinically evident level I, II axillary lymph node metastases
N3	Metastases in ipsilateral infraclavicular (level III axillary) lymph node(s) with or without level I, II axillary lymph node involvement; or in clinically detected ipsilateral internal mammary lymph node(s) with clinically evident level I, II axillary lymph node metastases; or metastases in ipsilateral supraclavicular lymph node(s) with or without axillary or internal mammary lymph node involvement
N3a	Metastasis in ipsilateral infraclavicular lymph node(s)
N3b	Metastasis in ipsilateral internal mammary lymph node(s) and axillary lymph node(s)
N3c	Metastasis in ipsilateral supraclavicular lymph node(s)
Pathologic
Nx	Regional lymph nodes cannot be assessed
N0	No regional lymph node metastasis histologically
N1	Micrometastases; or metastases in 1 - 3 axillary lymph nodes; and/or in internal mammary nodes with metastases detected by sentinel lymph node biopsy but not clinically detected
N1 mic	Micrometastases (greater than 0.2 mm and/or more than 200 cells, but none greater than 2.0 mm)
N1a	Metastases in 1 - 3 axillary lymph nodes, at least one metastasis greater than 2.0 mm
N1b	Metastases in internal mammary nodes with micrometastases or macrometastases detected by sentinel lymph node biopsy but not clinically detected
N1c	Metastases in 1 - 3 axillary lymph nodes and in internal mammary lymph nodes with micrometastases or macrometastases detected by sentinel lymph node biopsy but not clinically detected
N2	Metastases in 4 - 9 axillary lymph nodes; or in clinically detected internal mammary lymph nodes in the absence of axillary lymph node metastases
N2a	Metastases in 4 - 9 axillary lymph nodes (at least one tumor deposit greater than 2.0 mm)
N2b	Metastases in clinically detected internal mammary lymph nodes in the absence of axillary lymph node metastases
N3	Metastases in 10 or more axillary lymph nodes; or in infraclavicular (level III axillary) lymph nodes; or in clinically detected ipsilateral internal mammary lymph nodes in the presence of one or more positive level I, II axillary lymph nodes; or in more than three axillary lymph nodes and in internal mammary lymph nodes with micrometastases or macrometastases detected by sentinel lymph node biopsy but not clinically detected; or in ipsilateral supraclavicular lymph nodes
N3a	Metastases in 10 or more axillary lymph nodes (at least one tumor deposit greater than 2.0 mm); or metastases to the infraclavicular (level III axillary lymph) nodes
N3b	Metastases in clinically detected ipsilateral internal mammary lymph nodes in the presence of one or more positive axillary lymph nodes; or in more than three axillary lymph nodes and in internal mammary lymph nodes with micrometastases or macrometastases detected by sentinel lymph node biopsy but not clinically detected
N3c	Metastasis in ipsilateral supraclavicular lymph nodes

This classification remains a common clinical problem. Often, the clinical extent of disease is described numerically, as in the pathological classification. This confusion in description is a barrier in daily practice for a correct management disease. Pathological criteria are considered a reproducible and objective basis for treatment selection [8], but a suboptimal and imprecise clinical extent of disease can translate in a consequent under- or over-treatment. Besides the physical examination, which presents a considerable inter-subject variability, the method most widely used to clinically analyze the positivity of lymph nodes remains currently the ultrasound. However it seems reasonable in doubtful cases to perform a fine needle aspiration or lymph node biopsy, in a second instance. The role of MRI is still controversial.

## Clinical Evidence

In the last few decades, breast cancer management has greatly progressed, international guidelines have been provided to standardize clinical approach, albeit differences in multidisciplinary decision treatment still remain. Before the neo-adjuvant chemotherapy era, the surgical approach provided therapeutic and diagnostic elements to the subsequent adjuvant treatment; nowadays the employment of induction chemotherapy has confused the selection criteria for adjuvant RT. Approximately 80-90% of patients submitted to preoperative chemotherapy obtain at least a partial response to treatment [5, 6]. This evidence implicates that for 80-90% of patients the pathologic stage after neo-adjuvant treatment is different from the clinical stage at diagnosis. It is not well defined if this change in stage disease will influence the overall survival. Certainly it is recommended that the RT should be made by pre-treatment clinical stage, regardless of response to chemotherapy, because trials have shown that it offers clear clinical advantage [9]. Buchholz et al [10] have demonstrated a loco-regional free survival advantage for those patients who received RT, regardless of a pathological disease reduction after neo-adjuvant chemotherapy. They asserted that the response to induction chemotherapy does not reduce the risk of local recurrence; on the contrary it is a function of both clinical and pathological stage. Huang et al [4] reported a significantly local regional control advantage in those patients who received RT adjuvant versus who did not receive it (89% vs. 69%). McGuire et al [11] concluded that RT provided a significant clinical benefit, even in the outcome of patients who achieved a pathologic complete response to neo-adjuvant chemotherapy. Table 2 presents data concerning the RT and outcomes details for patients with clinical locally advanced disease and a subsequent pathological downstage to neo-adjuvant chemotherapy. Although these trials were not randomized, the 13th St Gallen International Breast Cancer Conference Expert Panel recommended radiotherapy solely based on clinical stage disease [9]. Regional lymph node status at diagnosis remains one of the strongest predictors of long-term prognosis in breast cancer [12]. No randomized studies exist and no ongoing trials explore the role of induction chemotherapy in adjuvant RT indication [13]. A randomized trial will probably define the potential protective role of induction chemotherapy in these patients.

**Table 2:** Clinical Outcomes According to Use of Radiation Therapy

Author	10-year local recurrence			10-year overall survival		
	No RT (%)	RT (%)	P value	No RT (%)	RT (%)	P value
Huang et al [4]	59	16	< 0.0001	47	54	0.063
Buchholz et al [10]*	53	23	< 0.001	-	-
McGuire et al [11]	33.3	7.3	0.04	77.3	33.3	0.0016

*Data refer to 5-year local recurrence.

## Current Standard of Care for Patients With Locally Advanced Disease

The current standard treatment approach for patients presenting with locally advanced breast cancer is induction chemotherapy followed by surgery and adjuvant RT. Adjuvant RT is always indicated, independent of response to initial chemotherapy and/or surgical approach, due to its role in optimizing local disease control. This recommendation is based on the results from major clinical trials conducted over the past 20 years, in which a mortality reduction of 5% was proven [14].

## Conclusion

According to international recommendations, treatment strategy should be decided by a multidisciplinary team. In locally advanced breast cancer, the efficacy of induction chemotherapy does not influence the efficacy of adjuvant RT. RT should be recommended in this subgroup of patients despite their response to neo-adjuvant treatment.
